# The Value of Preoperative Alpha-L-Fucosidase Levels in Evaluation of Malignancy and Differential Diagnosis of Urothelial Neoplasms

**DOI:** 10.1155/2020/6723616

**Published:** 2020-07-25

**Authors:** Dongshan Chen, Naidong Xing, Zhanwu Cui, Cong Zhang, Zhao Zhang, Dawei Li, Lei Yan

**Affiliations:** ^1^Department of Urology, Qilu Hospital of Shandong University, Wenhuaxi Road 107#, Jinan 250012, China; ^2^Department of Urology, Second Traditional Chinese Medicine Hospital of Dezhou City, Zhongxing Road 245#, Dezhou 253500, China

## Abstract

**Purpose:**

To evaluate the role of Alpha-L-fucosidase (AFU) in diagnosis and differential diagnosis of pure urothelial carcinoma (UC), urothelial carcinoma with squamous differentiation (UCSD), and squamous cell carcinoma (SqCC).

**Methods:**

A retrospective study was performed for 599 patients who were histologically confirmed with urothelial tumor. Preoperative AFU levels were compared across the distinct subgroups with different clinicopathological parameters. ROC curve analysis and logistic regression analysis were performed to further evaluate the clinical application value of serum AFU levels in diagnosis and differential diagnosis of urothelial tumors.

**Results:**

There were no statistically significant differences in the AFU levels between different groups with different malignant degrees (UC versus papilloma and papillary urothelial neoplasm of low malignant potential [PUNLMP], high-grade UC versus low-grade UC, invasive versus noninvasive malignant uroepithelial tumor) and different pathological types (UC, UCSD, and SqCC) (all *P* > 0.05). ROC curve analysis and logistic regression analysis showed that there was no statistically significant association between AFU levels and the tumor characteristics (all *P* > 0.05).

**Conclusions:**

Preoperative AFU levels cannot serve as a reliable predictor for malignant degree and differential diagnosis, including pure UC, UCSD, and SqCC of urothelial tumors.

## 1. Introduction

Malignant uroepithelial tumor (MUT) is a common malignant tumor in the genitourinary system and can be categorized as urothelial and nonurothelial carcinomas (nUC). Although the majority of malignancy are urothelial in histology, variants or divergent differentiations, such UCSD, exist [1, 2], which poses a great diagnostic and treatment challenge. In addition, nonurothelial carcinomas are relatively rare, and the most common subtypes of those include SqCC, adenocarcinoma and neuroendocrine tumors [3]. However, previous research has shown that nUC confer a worse prognosis and are more resistant to conventional treatment [4]. The differential diagnosis of pure UC (UC without divergent differentiations), UCSD, and SqCC relies not only on histopathological features, but also on sufficient precedent pathology and medical history taking [5]. Therefore, there is an urgent need to find a sensitive biological marker to identify SqCC, UC, and UCSD and evaluate the malignant degree of them.

Fucosylated glycan, such as blood type A, B, and H antigens and various Lewis antigens, is of crucial importance in a variety of physiological and pathological processes, including tissue development, infection, inflammation, and tumor metastasis [6]. The variation of fucosylated glycan both in quantity and in structure had been observed in a number of cancers [7]. Alpha-L-fucosidase (AFU), a liposomal enzyme involving the degradation of various fucosylated glycans, has been generally accepted as tumor markers which are correlated with early diagnosis and prognosis of hepatic carcinoma and colorectal cancer [8, 9]. Additionally, alpha-L-fucosidase-1 was reported to be a useful marker to distinguish mucoepidermoid carcinoma from oral squamous cell carcinoma [10]. However, the clinical application value of AFU in the MUT is unclear up to date. In this study, we mainly investigated the association between serum AFU levels and clinicopathological characteristics of MUT patients and explored the diagnostic significance of circulating AFU levels in patients with SqCC, UC, and UCSD.

## 2. Materials and Methods

### 2.1. Patient Data

A retrospective study was performed for 599 patients who were histologically confirmed with urothelial tumor and underwent operation at the Department of Urology, Qilu Hospital of Shandong University, between July 2014 and March 2018. Among 599 patients, 588 patients were diagnosed with urothelial tumor, and 11 patients were diagnosed with SqCC. The exclusion criteria for all patients in this study were as follows:

1. Coexisting any other malignancy
2. History of malignant tumor, including MUT and other malignancies
3. Patients receiving any adjuvant treatments, such as radiotherapy or chemotherapy before surgery
4. Patients with inadequate clinical information

### 2.2. Data Collection

The clinical information including patient age at the time of diagnosis, sex, smoking history, routine blood examination results (white blood cell count, platelet count, plasma fibrinogen level, etc.), and corresponding tumor characteristics were obtained from the electronic patient records at our institution. Tumor grade was assessed according to the 1998 WHO/ISUP classification. However, the postoperative pathology results collected in clinical practice could not provide sufficient information for clinicopathologic stage.

### 2.3. AFU Measurement

Prior to any clinical interventions, venous blood of patients was collected in the early morning after 12 hours of fast and was stored in test tubes with separating gel and coagulant. Subsequently, the serum AFU activity was detected by The Roche Cobas 8000 automatic analyzer. The tests were carried out complying with the standard operating procedure.

### 2.4. Statistical Analysis

The Statistical Package for Social Science version 22.0 (SPSS Inc, Chicago, IL, USA) was used for statistical analyses and data was presented as mean ± standard deviation (SD). The normal distribution was assessed by the Kolmogorov–Smirnov test. Normal distribution data was compared by the student tests, otherwise by the Mann–Whitney *U* test or Kruskal–Wallis H-test for two or more than two groups, respectively. *P* values <0.05 in two-tailed tests were considered statistically significant.

## 3. Results

### 3.1. Clinical Characteristics of Study Population

**Figure 1:**
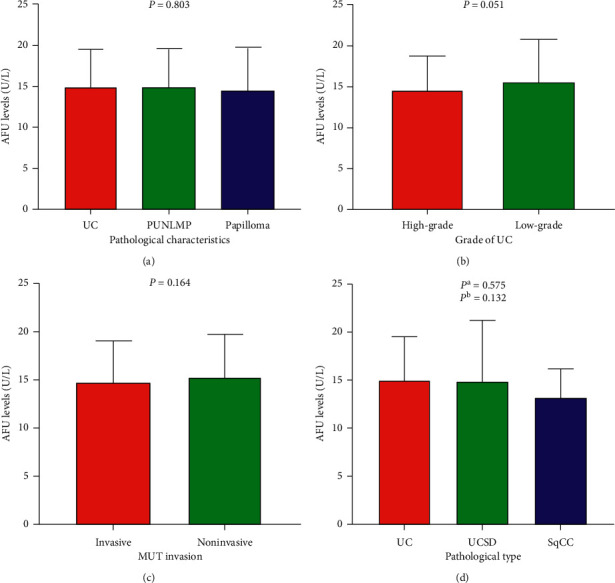
Comparison of AFU levels (a) among UC, PUNLMP, and Papilloma patients and (b) between high-grade and low-grade UC patients; (c) between invasive and non-invasive patients; (d) among pure UC, UCSD, and SqCC.

There were a total of 599 patients with newly diagnosed uroepithelial tumor enrolled in this study, which included 579 pure urothelial tumor. In addition, 455 were men and 144 were women, with a median age at diagnosis of 65 years ranging from 17 to 94. The mean preoperative AFU level of all patients was 14.94 ± 4.53 U/L. We found that preoperative AFU levels were significantly correlated with sex, smoking history, painless macroscopic hematuria, white blood cell (WBC), and lactate dehydrogenase (LDH) (all *P* < 0.05). In the cohort of UC, the mean preoperative AFU level of patients with pure UC (14.99 ± 4.50) was higher than that in patients with PUNLMP (14.93 ± 4.67 U/L) and papilloma (14.56 ± 5.22 U/L), but with no statistical significance (*P*=0.803, Figure 1). Besides, there was no obvious statistical significance in AFU levels between early-stage and advanced-stage disease (high-grade versus low-grade pure UC: 14.64 ± 4.10 versus 15.67 ± 5.12 U/L, *P*=0.051; invasive MUT versus noninvasive MUT: 14.79 ± 4.27 versus 15.38 ± 4.36 U/L, *P*=0.164, Figure 1). The other clinical characteristics of enrolled patients are presented in Table 1.

**Figure 2:**
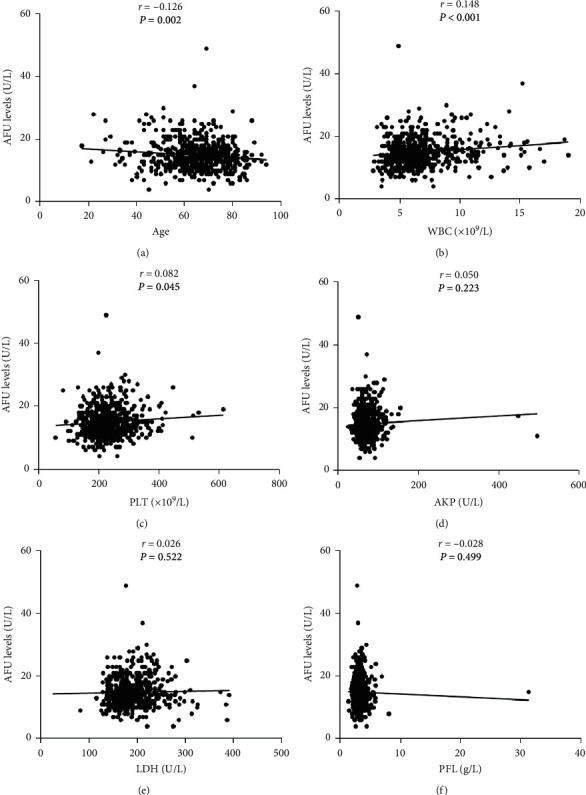
Linear correlations between AFU levels and age, WBC, PLT, AKP, LDH, and PFL in urothelial tumor patients.

Linear correlation analyses were performed to further evaluate correlations between AFU levels and clinical parameters in urothelial tumor. The result showed that there was a weak linear correlation between age, WBC, platelet (PLT) and AFU levels (*r* = −0.126, *P*=0.002; *r* = 0.148, *P* < 0.001; *r* = 0.082, *P*=0.045, respectively) (Figures 2(a)–2(c)). However, alkaline phosphatase (AKP), LDH, and plasma fibrinogen (PFL) did not show any correlation with AFU levels (all *P* > 0.05, Figures 2(d)–2(f)).

### 3.2. Associations between AFU Levels and Pathological Characteristics of MUT Patients

To further evaluate the diagnostic value of AFU in MUT, the levels of serum AFU were studied and compared in pure UC, UCSD, and SqCC. Among 535 MUT patients, 11 were SqCC and 524 were UC, including 9 with UCSD. We found that the gradation from high to low according to the levels of serum AFU was pure UC (14.99 ± 4.50), UCSD (14.78 ± 6.44), and SqCC (13.15 ± 3.03), with no statistical significance (*P*=0.280, Table 2 and Figure 1(d)).

### 3.3. Diagnostic Value of Preoperative AFU Levels in Urothelial Tumor

**Figure 3:**
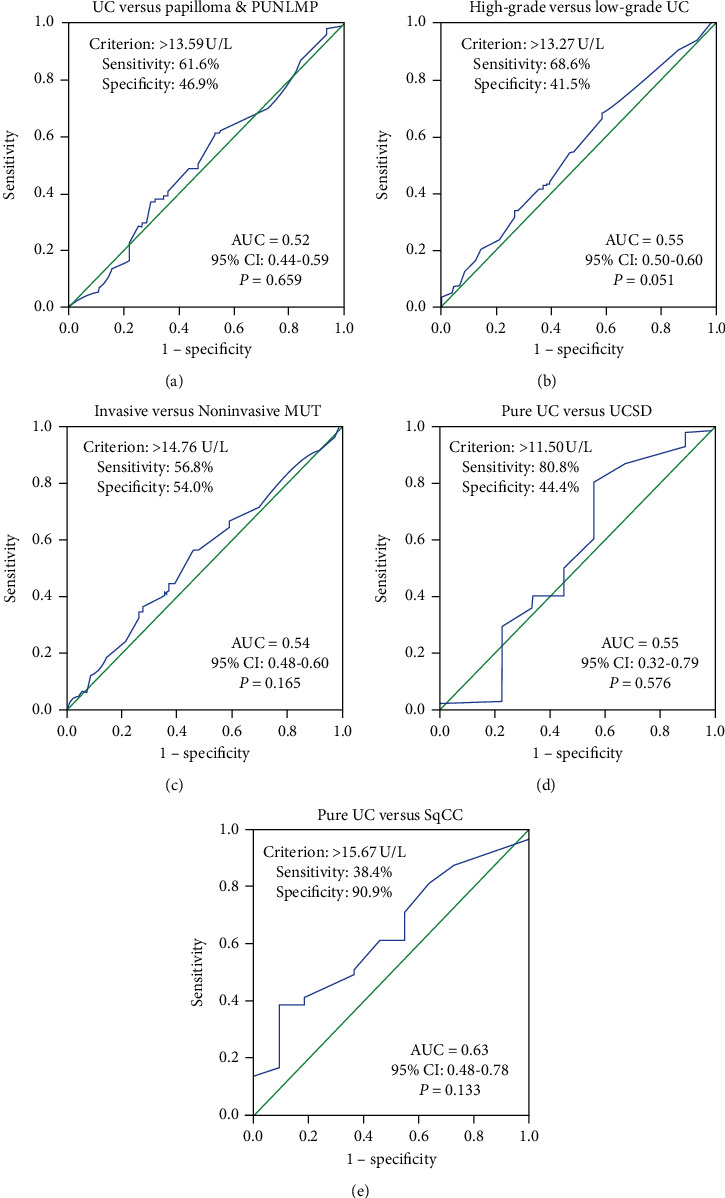
ROC curves for determination of cut-off value of AFU levels regarding prediction of (a) UC, (b) high-grade UC, (c) invasive MUT, (d) UCSD, and (e) SqCC.

The receiver operating characteristic (ROC) curves analysis for preoperative AFU levels was used to evaluate its value of qualitative diagnosis in urothelial tumor. The areas under the ROC curve (AUC) were 0.52 (95% CI 0.44–0.59, UC versus papilloma and PUNLMP), 0.55 (95% CI 0.50–0.60, high-grade UC versus low-grade UC), 0.54 (95% CI 0.48–0.60, invasive versus noninvasive MUT), 0.55 (95% CI 0.32–0.79, pure UC versus UCSD), and 0.63 (95% CI 0.48–0.78, pure UC versus SqCC), respectively. The corresponding optimal threshold values were 13.59 U/L, 13.27 U/L, 14.76 U/L, 11.50 U/L, and 15.67 U/L. However, all of *P* values were greater than 0.05, indicating that there was no clinical value of AFU levels in differential diagnosis and evaluation of malignancy extent of MUT. The sensitivity, specificity, cut-off value, and exact *P* value were shown in Figure 3.

Subsequently, logistic regression model of univariate and multivariate analysis was used to further evaluate the clinical impact of preoperative AFU levels on the prediction of histopathology. The univariate analysis showed that AFU levels were not significantly correlated with the differential diagnosis of urothelial tumor (UC and papilloma and PUNLMP, pure UC vs. UCSD, pure UC vs. SqCC; all *P* > 0.05; Table 3). Additionally, hematuria shows significant predictability on pathology results (UC versus Papilloma and PUNLMP: HR = 2.640, *P* < 0.001; pure UC versus SqCC: HR = 3.591, *P*=0.037; Table 3). By multivariable analysis, AFU levels were not independent predictors of pathologic diagnosis (all *P* > 0.05, Table 4). However, hematuria was an independent risk factor for UC and UCSD (UC versus Papilloma and PUNLMP: HR = 2.154, *P*=0.007; pure UC versus UCSD: HR = 4.761, *P*=0.007; Table 4).

## 4. Discussion

Although most of the neoplasms located in the urinary tract are mainly histologically urothelial carcinomas, the variation of morphological characteristics exists within tumors [11]. The most common variation type is squamous differentiation, which is characterized by the presence of keratin pearls, intercellular bridges, or both [12, 13]. Previous studies have shown that squamous differentiation is more resistant to radiotherapy, chemotherapy, and immunotherapy and possesses the characteristics of high risk of recurrence and poor prognosis as an independent prognostic factor of UC [2, 14, 15]. In addition, SqCC, as the most common variant in nonurothelial carcinomas, is usually discovered and diagnosed at its advanced stage, while a considerable number of patients have lymph node metastasis in neoplasms resection [5, 16]. Currently, there are limited clinical approaches for the early diagnosis and treatment of MUT. Therefore, there is a pressing need to find a specific, high sensitivity marker to improve differential diagnosis rate of pure UC, UCSD, and SqCC and predict the degree of malignancy, development trend, and prognosis.

As we know, AFU extensively exists in human cells and is continuously released into blood to circulate as a product of cell metabolism [17]. Human AFU is a hydrolase with particular biological and medical interest, whose main role is to hydrolyze the glycans participated in diverse interactions between cells and extracellular matrix [18, 19]. Glycosylation is an important physiological and pathological process, and abnormality of content and function of fucosylated glycan are common features of malignant neoplastic transformation, including tumor invasion and metastases, and other human diseases [20, 21]. The decreased or increased activities of serum AFU are commonly used as a diagnostic biomarker for fucosidosis and hepatocellular carcinoma, respectively [22, 23]. And constantly elevated AFU level in patients with liver cirrhosis contributes to early detection of hepatocellular carcinoma [24]. Besides, many studies on the relationships between the serum form of AFU and other diseases had been reported. The alterations of AFU activity and properties also possess a high clinical value in the diagnosis of colorectal cancer [25], ovarian cancer [26], chronic inflammation, and autoimmune disorders [27]. Moreover, a comprehensive research project that contains 188,077 patients with 64 clinically defined diseases and 9,519 healthy controls showed that serum AFU activities were higher in patients with preeclampsia, liver cancer, hepatitis, psoriasis, and multiple myeloma, while serum AFU activities were the lowest in patients with uremia, azotemia, myeloproliferative disorder, and Alzheimer's disease among the 64 diseases studied [28]. Although serum AFU is potentially valuable indicators for many cancers and many different types of diseases, investigation about its serum levels in a cohort of patients with urinary tract neoplasms is still lacking.

In the present study, we analyzed preoperative AFU levels and clinicopathological features of 599 patients with urothelial tumor. There were no statistically significant differences in the AFU levels between different groups with different malignant degrees (UC versus papilloma versus PUNLMP, *P*=0.803; high-grade versus low-grade UC, *P*=0.051; invasive versus noninvasive MUT, *P*=0.164). In addition, we did not find a statistically significant difference in AFU levels among distinct pathologic type (pure UC versus UCSD versus SqCC, *P*=0.280). Interestingly, we found that AFU levels were closely related to sex, smoking history, painless macroscopic hematuria, WBC, and LDH (all *P* < 0.05). Next, linear correlation analyses were performed and showed that only age, WBC, and PLT were related slightly to AFU levels (*r* = −0.126, *P*=0.002; *r* = 0.148, *P* < 0.001; *r* = 0.082, *P*=0.045, respectively). However, the correlation coefficients were low; they had no medical significance. To further evaluate the practical application value of AFU levels in diagnosis and differential diagnosis of urothelial tumor, ROC curves were performed for preoperative AFU levels regarding the prediction of pure UC, high-grade UC, invasive MUT, UCSD, and SqCC. AUC were 0.52 (UC versus papilloma and PUNLMP), 0.55 (high-grade UC versus low-grade UC), 0.54 (invasive versus noninvasive MUT), 0.55 (pure UC versus UCSD), and 0.63 (pure UC versus SqCC (all *P* > 0.05)), respectively, which indicated that AFU levels could not serve as a valuable indicator to reflect the extent of malignancy and pathological type of urothelial tumor. Furthermore, univariate and multivariate analysis further strengthened the evidence that serum AFU level was not an independent predictor of pure UC, UCSD, and SqCC. Interestingly, multivariate logistic regression analysis showed that painless macroscopic hematuria was an independent risk factor for UC and UCSD (UC versus Papilloma and PUNLMP: HR = 2.154, *P*=0.007; pure UC versus UCSD: HR = 4.761, *P*=0.035).

However, there are several limitations in the present study. Firstly, because the number of patients with UCSD and SqCC was relatively small and this is a retrospective, observational study, unknown sources of bias may exist. Secondly, all clinicopathological information was collected from a single institution; the universal conclusions of the study may be subject to certain restrictions.

In conclusion, there was no direct correlation between the levels of serum AFU and the tumor characteristics (tumor number, tumor size, and pathological characteristics (all *P* > 0.05)) in statistics. Thus, preoperative AFU levels cannot serve as a reliable predictor for malignant degree and differential diagnosis, including pure UC, UCSD, and SqCC, of urothelial tumor.

## Figures and Tables

**Table 1:** Correlations between preoperative AFU levels and clinicopathological parameters.

Variables	No. of patients (%)	AFU levels (U/L, mean ± SD)	*P* value
Patients	599	14.94 ± 4.53	

*Age* ^*a*^			
≤65y	306	15.30 ± 4.62	0.100^#^
＞65y	293	14.57 ± 4.40	

*Sex*			
Male	455	15.24 ± 4.61	**0.001** ^#^
Female	144	13.99 ± 4.11	

*Smoking history*			
Ever	220	15.42 ± 4.85	**0.049** ^#^
Never	379	14.66 ± 4.30	

*Painless macroscopic hematuria*			
Yes	429	15.18 ± 4.34	**0.010** ^#^
No	170	14.34 ± 4.92	

*Tumor number*			
Single	405	14.81 ± 4.59	0.372^#^
Multiple	194	15.23 ± 4.38	

*Tumor size* ^*ab*^			
≤2.8 cm	308	15.11 ± 4.75	0.229^#^
＞2.8 cm	291	14.77 ± 4.28	

*WBC* ^*a*^			
≤6.25 × 10^9^/L	301	14.54 ± 4.63	**0.005** ^#^
＞6.25 × 10^9^/L	298	15.35 ± 4.38	

*PLT* ^*a*^			
≤226 × 10^9^/L	305	14.68 ± 4.76	0.100^#^
＞226 × 10^9^/L	294	15.22 ± 4.26	

*AKP* ^*a*^			
≤68 U/L	316	14.73 ± 4.61	0.115^#^
＞68 U/L	283	15.19 ± 4.42	

*LDH* ^*a*^			
≤188 U/L	305	14.57 ± 4.42	**0.021** ^#^
＞188 U/L	294	15.33 ± 4.61	

*PFL* ^*a*^			
≤3.12 g/L	302	15.22 ± 4.68	0.115^#^
＞3.12 g/L	297	14.66 ± 4.36	

*Pathological characteristics*			
Papilloma	8	14.56 ± 5.22	0.803^*∗*^
PUNLMP	56	14.93 ± 4.67	
Pure UC	515	14.99 ± 4.50	

*Grade of pure UC*			
High-grade	340	14.64 ± 4.10	0.051^#^
Low-grade	175	15.67 ± 5.12	

*Tumor invasion of MUT*			
Invasive	328	14.79 ± 4.27	0.164^#^
Noninvasive	118	15.38 ± 4.36	
Unknown	89	—	

Continuous variables are expressed as median^a^. Bold values are statistically significant (*P* < 0.05).PUNLMP papillary urothelial neoplasm of low malignant potential; UC: urothelial cancer; MUT: malignant uroepithelial tumor; WBC: white blood cell; PLT platelet; AKP: alkaline phosphatase; LDH: lactate dehydrogenase; PFL: plasma fibrinogen. *P*^*∗*^: Kruskal–Wallis H-test; *P*^#^: Mann–Whitney *U* test.

**Table 2:** The level of serum AFU in patients with pure UC, UCSD, and SqCC.

MUT	No. of patients (%)	SA levels (mg/dL, mean ± SD)	*P* value
Pure UC	515	14.99 ± 4.50	0.575^a#^
UCSD	9	14.78 ± 6.44	0.132^b#^
SqCC	11	13.15 ± 3.03	0.280^*∗*^

Bold values are statistically significant (*P* < 0.05). *P*^*∗*^: Kruskal–Wallis H-test; *P*^#^: Mann–Whitney *U* test; P^a^: pure UC versus UC with squamous differentiation; P^b^: pure UC versus SqCC; P^c^: pure UC versus UC with squamous differentiation versus SqCC.

**Table 3:** Univariate analysis of preoperative variables prognostic for pure UC, UCSD, and SqCC.

Variables	UC versus papilloma and PUNLMP		Pure UC versus UCSD		Pure UC versus SqCC	
	HR (95% CI)	*P*	HR (95% CI)	*P*	HR (95% CI)	*P*
Sex	0.982 (0.531∼1.813)	0.953	0.601 (0.148∼2.440)	0.476	0.172 (0.049∼0.597)	**0.006**
Hematuria	2.640 (1.554∼4.485)	**0.000**	3.740 (0.989∼14.140)	0.052	3.591 (1.078∼11.963)	**0.037**
Tumor size	4.593 (2.395∼8.808)	**0.000**	3.302 (0.679∼16.046)	0.139	1.651 (0.477∼5.708)	0.428
WBC	1.120 (0.666∼1.883)	0.670	1.315 (0.349∼4.952)	0.686	4.733 (1.013∼22.120)	**0.048**
LDH	1.395 (0.827∼2.354)	0.212	2.170 (0.537∼8.770)	0.277	1.106 (0.333∼3. 670)	0.869
AFU level (>13.59^*∗*^ versus ≤ 13.59)	1.413 (0.838∼2.381)	0.195	1.281 (0.340∼4.827)	0.715	1.334 (0.402∼4.430)	0.638
AFU level (>11.50^*∗*^ versus ≤ 11.50)	1.031 (0.530∼2.005)	0.928	3.362 (0.886∼12.748)	0.075	2.401 (0.689∼8.363)	0.169
AFU level (>15.67^*∗*^ versus ≤ 15.67)	1.192 (0.691∼2.058)	0.527	1.249 (0.309∼5.052)	0.755	6.246 (0.793∼49.167)	0.082

Bold values are statistically significant (*P* < 0.05).HR: hazard ratio; 95% CI 95% confidence interval.

**Table 4:** Multivariate analysis of preoperative variables prognostic for pure UC, UCSD, and SqCC.

Variables	UC versus papilloma and PUNLMP		Pure UC versus UCSD		Pure UC versus SqCC	
	HR (95% CI)	*P*	HR (95% CI)	*P*	HR (95%CI)	*P*
Sex	1.080 (0.564∼2.070)	0.816	0.968 (0.220∼4.256)	0.965	0.192 (0.050∼0.740)	**0.016**
Hematuria	2.154 (1.237∼3.750)	**0.007**	4.761 (1.116∼20.303)	**0.035**	3.259 (0.880∼12.065)	0.077
Tumor size	4.374 (2.246∼8.517)	**0.000**	3.905 (0.769∼19.828)	0.100	1.475 (0.381∼5.706)	0.574
WBC	1.300 (0.744∼2.270)	0.357	1.584 (0.371∼6.765)	0.535	7.714 (1.504∼39.568)	**0.014**
LDH	1.525 (0.875∼2.658)	0.136	2.340 (0.537∼10.187)	0.257	2.020 (0.518∼7.879)	0.311
AFU level (>13.59^*∗*^ versus ≤ 13.59)	1.587 (0.913∼2.760)	0.102		n.d.		n.d.
AFU level (>11.50^*∗*^ versus ≤ 11.50)		n.d.	3.235 (0.792∼13.213)	0.102		n.d.
AFU level (>15.67^*∗*^ versus ≤ 15.67)		n.d.		n.d.	4.736 (0.573∼39.145)	0.149

Bold values are statistically significant (*P* < 0.05). HR: hazard ratio; 95% CI: 95% confidence interval; n.d.: not done.

## Data Availability

The data used to support the findings of this study are included within the supplementary information files.

## Supplementary Materials

Supplementary Materials The given supplementary material was the data used to support the findings of this study.
